# Dietary Carnosic Acid Supplementation Improves the Growth Performance, the Antioxidant Status, and Diversity of Intestinal Microbiota in Broilers

**DOI:** 10.3390/antiox14081026

**Published:** 2025-08-21

**Authors:** Sheng Zhang, Qin Wang, Jingjing Dong, Guanhuo Li, Kaiyuan Niu, Junhao Pan, Linghan Xia, Yibing Wang, Shouqun Jiang

**Affiliations:** Institute of Animal Science, Guangdong Academy of Agricultural Sciences, State Key Laboratory of Swine and Poultry Breeding Industry, Key Laboratory of Animal Nutrition and Feed Science in South China, Ministry of Agriculture and Rural Affairs, Guangdong Provincial Key Laboratory of Animal Breeding and Nutrition, Guangzhou 510640, China; zhangszin@163.com (S.Z.); wqsyyyzyq@163.com (Q.W.); 2112359015@stu.fosu.edu.cn (J.D.); 13422647014@163.com (G.L.); 17555180040@163.com (K.N.); pq220320@163.com (J.P.); xlh1708747261@163.com (L.X.)

**Keywords:** antioxidant capacity, broilers, carnosic acid, Keap1/Nrf2, intestinal microbiota

## Abstract

Carnosic acid (CA), a natural phenolic terpenoid compound, is widely distributed in plants such as sage and rosemary, and exhibits a strong antioxidant capacity. The aim of this study was to investigate the effects of different levels of CA on growth performance, antioxidant capacity, and intestinal health of yellow-feathered broilers, and then to determine the optimal dose of CA to promote sustainable broiler production. A total of 384 1-day-old yellow-feathered broilers were randomly allocated into six treatment groups with eight replicates per group and eight birds per replicate pen. The control group (CON) was fed a basal diet and the CA treated groups (CA5, CA10, CA20, CA40, and CA80) were fed diets given different doses of CA (5, 10, 20, 40, and 80 mg/kg), respectively, for 53 days (1~21 d and 22~53 d). The results showed that, in the later stages of the experiment, supplementation with 10, 20, and 40 mg/kg of CA increased (*p* < 0.05) the final body weight and average daily gain. Morphometric analyses of the jejunum showed that supplementation of CA increased (*p* < 0.05) the ratio of villus height to crypt depth (V/C). Antioxidant indices revealed that CA significantly reduced MDA levels in plasma, liver, and jejunum, while enhancing activities of GSH-Px, T-SOD, and T-AOC (*p* < 0.05). Moreover, CA upregulated hepatic *Nrf2*, *HO-1*, *GSH-Px*, and *GSR* expression via downregulated *Keap1*. The analysis of intestinal microbiota showed that CA increased (*p* < 0.05) microbial α diversity (Ace, Chao, and Sobs indices) and increased (*p* < 0.05) beneficial bacteria, such as *Streptococcus*, *Enterococcus*, and *Phascolarctobacterium*. In conclusion, CA improves growth performance, antioxidant capacity, intestinal health, and gut microbial diversity in broilers. Under the conditions of this experiment, quadratic regressions for different variables showed that the optimal range for supplemental CA in chicken’s diet was 19.11~76.85 mg/kg. Combined with experimental observation and regression analysis, the optimal level of supplementation was 40 mg/kg.

## 1. Introduction

Chicken has become an important part of the global meat market, ranking second only to pork in consumption [1]. Poultry production faces serious challenges after the total ban on antimicrobials in feed, such as reduced growth performance, impaired meat quality, and intestinal damage [2], which prompts the search for bioactive alternatives to antibiotics to maintain the health production in poultry. Currently, more and more natural compounds with antioxidant activity have shown beneficial effects in poultry production as promising antibiotic substitutes [3,4,5].

As natural and nutritionally bioactive substances, plant extracts play an important role in enhancing growth performance [6], alleviating oxidative stress [7], and improving intestinal health of poultry [8]. Among them, phenolic antioxidants demonstrate strong free radical-scavenging activity by donating hydrogen ion and electron via phenolic hydroxyl groups or chelating metal ions [9]. Phenolic compounds, with their remarkable antioxidant properties, can regulate the Keap1/Nrf2 signaling pathway through multiple targets, thereby exerting cytoprotective effects [10]. Carnosic acid (C_20_H_28_O_4_, CA), a phenolic terpenoid compound, is widely present in plants such as *Salvia* and rosemary (*Rosmarinus officinalis* L.), and exhibits multiple benefits in livestock and poultry nutrition, including the following: (1) enhancing antioxidant capacity by regulating the endogenous antioxidant system, including upregulating antioxidant-related genes and the activity of antioxidant enzymes [11]; (2) reducing the secretion of inflammatory factors and inhibiting the proliferation of harmful bacteria [12]; (3) improving meat quality by regulating fat metabolism [13]. Previous research has indicated that CA can improve the growth performance of broiler chickens, reduce diarrhea rate and mortality rate [14,15], enhance the reproductive performance and egg quality of breeder chickens [16], and alleviate cold/heat stress damage [17,18], yet findings on its efficacy and optimal dosage remain inconsistent. In this regard, further research is needed to evaluate the effects of CA on broilers, including its impacts on growth performance, meat quality, and antioxidant status, as well as to determine the most effective supplementation level in diets, further providing a basis for its potential application in broiler production.

## 2. Materials and Methods

### 2.1. Animal Experimental Design

A total of 384 1-day-old fast-growing, yellow-feathered broilers were randomly allocated into 6 treatments, with 8 replicates per treatment and 8 birds per replicate pen. Birds in the control group (CON) were fed a basal diet, and the other groups (CA5, CA10, CA20, CA40, and CA80) were fed diets supplemented with 5, 10, 20, 40, and 80 mg/kg of CA (85%, Shanghai Yuanye Bio-Technology Co., Ltd., Shanghai, China), respectively.

### 2.2. Diets and Chicken Husbandry

The diets were formulated as recommended by Chinese Nutrient Requirements of Yellow Chickens (NY/T3645-2020, Ministry of Agriculture PRC, 2020) [19]. The composition and nutrient content of diets are shown in Table 1. The experiment was carried out in the testing farm of Institute of Animal Science, Guangdong Academy of Agricultural Sciences. Birds were raised in floor pens with wood shavings litter for 53 d. Water and diets were provided ad libitum throughout.

### 2.3. Measurement of Growth Performance

Birds were weighed at d l, d 21, and d 53 on a pen basis, the average daily gain (ADG), average daily feed intake (ADFI), and feed to gain ratio (F/G) were calculated.

### 2.4. Sample Collection

At d 21 and d 53, after 12 h of feed-withdrawal, one bird close to average body weight (BW) per replicate was chosen. Blood (5 mL) was collected from the wing veins and drawn into vacutainers containing anticoagulant (BD™ Vacutainer, Franklin Lakes, NJ, USA). The blood samples were then centrifuged at 3500× *g* for 10 min at 4 °C to separate the plasma, which was subsequently stored at −80 °C until analysis. Then, the birds were euthanized and dissected for samples. For birds at 21 d, the duodenum, jejunum, ileum, liver, spleen, thymus, and bursa of Fabricius were collected. For birds at 53 d, the duodenum, jejunum, ileum, liver, spleen, cecum, and pectoral muscle were collected and the mid-jejunal segments were rinsed with sterile saline and then partially fixed by immersion in 4% paraformaldehyde for further morphological observation.

### 2.5. Calculation of Relative Weights of Immune Organs

The liver, spleen, thymus, and bursa of Fabricius of birds at 21 d and the liver and spleen of birds at 53 d were blotted and weighed. The relative weights (weight of organ/BW × 100%) were calculated.

### 2.6. Determination of Meat Quality

Pectoralis major muscles were blotted, and objective indicators of meat quality, including instrumental color as L*(lightness), a*(redness), and b*(yellowness) value, shear force, drip loss, and pH value, were determined at 45 min and 24 h post mortem, with the methods described previously by Wang et al. [20]. The rate of pectoral muscle was calculated as the weight of right pectoralis major muscle/BW × 100%. The intramuscular fat (IMF) content of the left pectoralis muscle was determined using the Soxhlet extraction method [21] and calculated using the following formula: IMF content = fat content/dry weight of muscle × 100%.

### 2.7. Determination of Antioxidant Variables

Samples of the liver, duodenum, jejunum, and ileum were homogenized with saline and centrifuged (12,000 rpm, 10 min, 4 °C) for clarified homogenates. The activities of glutathione peroxidase (GSH-Px), total superoxide dismutase (T-SOD), total antioxidant capacity (T-AOC), and catalase (CAT), and the concentration of MDA in the plasma, liver, duodenum, jejunum, and ileum were assayed with colorimetric kits (A001-3-2, A005-1-2, A015-1, A003-1-1, A007-1-1, Nanjing Jiancheng Institute of Bioengineering, Nanjing, PRC) and a Microplate Reader (Bio-Rad, Hercules, CA, USA). Results of the intestines were normalized to protein concentration, quantified by the BCA Protein Assay Kit (NCI3225 CH, Thermo Fisher Scientific, Waltham, MA, USA).

### 2.8. Intestinal Morphological Observation

The jejunum fixed with paraformaldehyde was dehydrated and embedded in paraffin (5 μm thick). After hematoxylin–eosin (H&E) staining, morphological images were scanned with a white light scanner (Hamamatsu Photonics Co., Ltd., Hamamatsu City, Japan); then the field of view was intercepted with NDP.view2.9.22 software. The villus height and crypt depth were measured using Image J software (version 2.1.4.7, Media Cybernetics, Bethesda, MD, USA), and the ratio of villus height to crypt depth (V/C) was calculated.

### 2.9. Real-Time PCR Analysis

The relative expressions of *GST*, *SOD-1*, *SOD-2*, *GSR*, *Keap1*, *HO-1*, *Nrf2*, and *GSH-Px* were determined by Real-time PCR. Total RNA was extracted from hepatic tissue using TRIzol™ reagent (Takara Bio, Tokyo, Japan), and was reverse-transcribed with the PrimeScript II 1st Strand cDNA Synthesis Kit (Takara, Tokyo, Japan). Real-time PCR was performed on the CFX 96 Real-time PCR Detection System (Bio-Rad, Hercules, CA, USA). The primer sequences used are shown in Table 2. The relative mRNA expression was calculated according to the 2^−ΔΔCt^ method, and data for each target transcript were normalized to the mRNA level of the *β-actin* gene.

### 2.10. 16S rRNA Gene Sequencing

Cecal contents were used to extract DNA using FastDNA™ Spin Kit for Feces (116570200, MP Biomedicals, Irvine, CA, USA). The V3 to V4 hypervariable region of the 16 S rRNA gene was amplified using 338 F (5′-ACTCCTACGGGAGGCAGCAG-3′) and 806 R (5′-GGACTACHVGGGTWTCTAAT-3′) primers. Purification of PCR products was performed with AxyPrep DNA Gel Extraction Kit (AP-GX-250 G, Axygen Scientific, Inc., Union City, CA, USA). After quantification of the purified pooled amplicon, NEXTFLEX^®^ Rapid DNA-Seq Kit (NOVA-5188-403, Bioo Scientific, Austin, TX, USA) was used to construct the library, which was then sequenced with Illumina NovaSeq PE250 system. Fastp 0.23.1 was applied to process and assess the quality of the raw 16 S sequence data, and Flash 1.2.7 was used to concatenate paired-end reads. UPARSE 7.1 was employed to quality-filter and cluster into operational taxonomic units (OTUs) at 97% similarity. RDP classifier 2.2 was used to annotate OTUs picking against the Silva 16 S rRNA database (v138). Then, OTU clustering, Venn diagrams, alpha diversity analyses, and community bar plots were obtained. The relative abundance of Kyoto Encyclopedia of Genes and Genomes (KEGG) Ortholog (KO) was obtained by Phylogenetic Investigation of Communities by Reconstruction of Unobserved State (PICRUSt2) against KO information through the OTUs greengenes ID.

### 2.11. Statistical Analysis

The experimental data were analyzed by one-way analysis of variance (ANOVA) in SPSS software (version 27.0), and when *p* < 0.05, multiple comparisons between treatments were performed using Duncan’s multiple range tests. The results were presented as the mean ± standard error (SEM). Differences were considered to be statistically significant at *p* < 0.05, and not statistically significant at *p* ≥ 0.05. Where appropriate, polynomial regressions were fitted to test for linear and quadratic effects in response to CA supplementation [22]. When a significant quadratic component was demonstrated (*p* < 0.05), regression analysis was used to estimate supplemental CA optimization.

## 3. Results

### 3.1. Growth Performance

The effects of supplementation with CA on the growth performance of broilers are shown in Table 3. From 1 to 21 days, supplementation with CA had no significant effects on the growth performance of broilers (*p* > 0.05). The final BW (at d 53) and ADG (from 22 to 53 and 1 to 53 days of age) were increased quadratically (*p* < 0.05). Compared with birds in the CON group, supplementation with 10, 20, and 40 mg/kg of CA significantly increased these three above variables. Furthermore, 80 mg/kg of CA decreased the survival rate of birds from 22 to 53 or 1 to 53 days of age (*p* < 0.05).

### 3.2. Relative Weights of Immune Organs

As shown in Table 4, supplementation with CA had no influence on relative weights of the liver, spleen, thymus, and bursa of Fabricius of broilers at 21 days of age. For birds at 53 d, there were both linear (*p* < 0.05) and quadratic (*p* < 0.05) effects of the supplemental CA on the relative weight of the liver. Broilers supplemented with 80 mg/kg of CA decreased (*p* < 0.05) the relative weight of liver, compared with that in the CON group.

### 3.3. Meat Quality

As shown in Table 5, dietary supplementation with CA had no significant effect on the objective indicators of meat quality. Dietary supplementation with CA reduced the IMF content in the pectoral muscle quadratically (*p* < 0.05) and, compared with birds in the CON group, 40 mg/kg of CA reduced the IMF content of broilers (*p* < 0.05).

### 3.4. Jejunal Morphology

**Figure 1:**
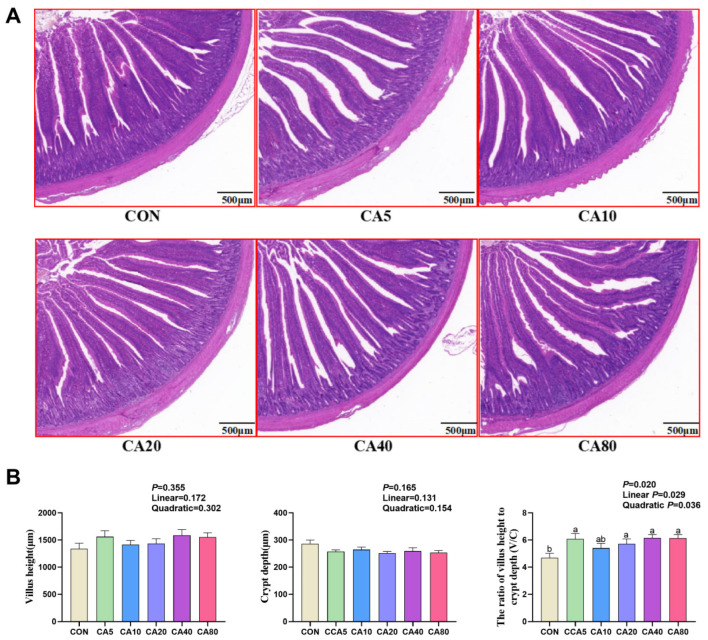
Effects of carnosic acid on jejunal morphology of 53-day-old broilers. (**A**) Hematoxylin–eosin (H&E) stained jejunum. Scale bar at 500 μm. (**B**) Villus height, crypt depth, and the ratio of villus height to crypt depth (V/C). ^ab^ Means with common superscripts within a row do not differ significantly (*p* ≥ 0.05). CON: basal diet; CA5/10/20/40/80: basal diet supplemented with 5, 10, 20, 40, and 80 mg/kg of carnosic acid.

The morphological structure of the jejunum by H&E staining is shown in Figure 1A. The intestinal morphology of each group was intact without obvious damage. Statistical results (Figure 1B) showed that dietary supplementation with CA had both linear and quadratic effects on the jejunal V/C of broilers (*p* < 0.05), and supplementation with 5, 20, 40, and 80 mg/kg of CA increased the V/C ratio compared with the control group (*p* < 0.05).

### 3.5. Antioxidant Capacity

As shown in Table 6, for the plasma, supplemental CA showed both linear and quadratic effects (*p* < 0.05) on activities of GSH-Px, T-SOD, and T-AOC. Compared with birds in the CON group, supplementation with CA increased (*p* < 0.05) the activities of GSH-Px (CA80) and T-AOC (CA10 and CA80). For the liver, compared with the CON group, supplemental CA significantly decreased the MDA level (CA80), and increased the CAT activity (CA5 and CA10). For the duodenum, supplemental CA showed both linear and quadratic effects (*p* < 0.05) on GSH-Px and T-SOD activities. Specifically, compared with birds in the CON group, supplementation with CA increased (*p* < 0.05) the activities of GSH-Px (CA10, CA20, CA40, and CA80), T-AOC and T-SOD (CA5, CA10, CA40, and CA80). For the ileum, supplemental CA showed both linear and quadratic effects (*p* < 0.05) on GSH-Px and T-AOC activities; compared with the CON group, birds in CA5, CA10, CA20, and CA40 showed higher GSH-Px activity.

As shown in Table 7, for 53-d broilers, there were quadratic effects of supplemental CA on MDA level in the plasma, liver, and jejunum (*p* < 0.05). In detail, compared with birds in the CON group, supplementation with CA decreased (*p* < 0.05) MDA levels in the plasma (CA20, CA40, and CA80), the liver (all CA groups), and the jejunum (CA5 and CA80). Also, CA increased (*p* < 0.05) the activity of T-SOD (CA40 and CA80) in the liver, as well as the activity of GSH-Px (CA5, CA10, and CA20) and CAT (CA20 and CA80) in the ileum.

### 3.6. The Relative Expression of Antioxidative Genes

**Figure 2:**
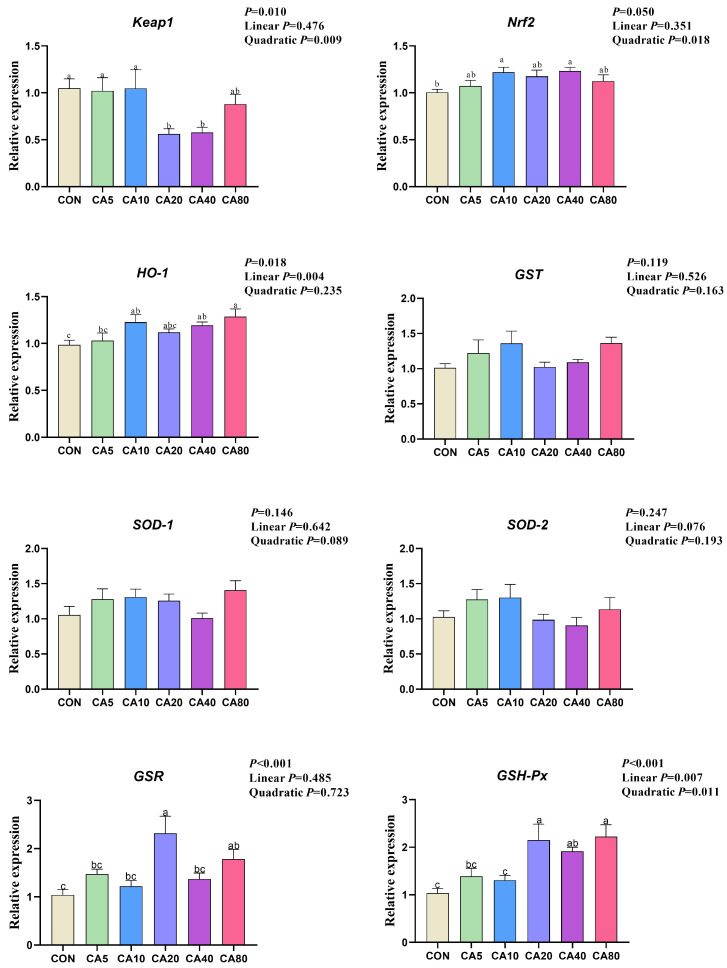
Effects of carnosic acid on relative expression of antioxidant genes in the liver of 53-day-old broilers. ^a–c^ Means with common superscripts do not differ significantly (*p* ≥ 0.05). CON: basal diet; CA5/10/20/40/80: basal diet supplemented with 5, 10, 20, 40, and 80 mg/kg of CA; *SOD-1*: superoxide dismutase 1; *SOD-2*: superoxide dismutase 2; *GST*: glutathione s-transferase; *GSR*: glutathione reductase; *GSH-Px*: glutathione peroxidase; *Keap1*: kelch-like ECH-associated protein-1; *Nrf2*: nuclear factor erythroid 2-related factor 2; *HO-1*: heme oxygenase-1.

As shown in Figure 2, for 53-d broilers, supplemental CA affected the relative expression of *Keap1* (quadratic), *Nrf2* (quadratic), and *HO-1* (linear) in the liver. Compared with birds in the CON group, supplementation with CA significantly decreased the relative expression of *Keap1* (CA20 and CA40), and increased the relative expression of *Nrf2* (CA10 and CA40) and *HO-1* (CA10, CA40 and CA80) in the liver. There were also significant effects of the supplemental CA on the relative expression of *GSH-Px* (linear and quadratic) and *GSR* in the liver. In detail, compared with birds in the CON group, supplementation with CA significantly increased the relative expression of *GSR* (CA20 and CA80) and *GSH-Px* (CA20, CA40, and CA80).

### 3.7. Regression Analysis of Optimal Supplemental Level of CA Based on Quadratic Regression

The optimal levels of supplemental CA on yellow-feathered broilers from the quadratic regressions (the maximum response from a quadratic model) are shown in Table 8. The optimal levels of CA supplementation were 40.53, 40.14, and 40.52 mg/kg for BW at 53 d, ADG (from 22 to 53 d), and ADG (from 1 to 53 d), respectively.

For 21-d broilers, the optimal supplemental levels of CA were 24.17 mg/kg for hepatic T-AOC, 74.61 mg/kg for duodenal T-SOD activity, 60.05 mg/kg for duodenal GSH-Px activity, and 30.81 mg/kg for ileal GSH-Px activity. For 53-d broilers, the optima was 19.11 mg/kg for relative weight of the liver, and 43.04 mg/kg for IMF in the pectoral muscle. Also, the optima was 57.28 mg/kg for the jejunal V/C, 55.23, 50.58, and 56.49 mg/kg for MDA levels in the plasma and liver, 71.47 mg/kg for T-SOD activity, and 45.02, 44.35, 76.85, and 61.58 mg/kg for relative expression of *Keap1*, *Nrf2*, *HO-1* and *GSH-Px* in the liver.

### 3.8. Intestinal Microbiota

**Figure 3:**
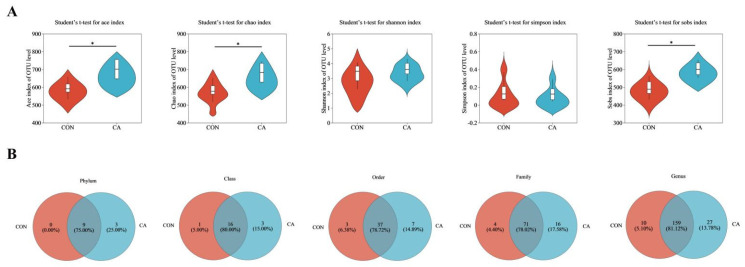
Effect of carnosic acid on α diversity and microbial composition of the cecum. (**A**) Alpha-diversity (Shannon, Chao, Ace, Sobs, Simpson). (**B**) Number of OTUs at the phylum, class, order, family, and genus level. Dates are shown as mean ± SEM (*n* = 8), * *p* < 0.05. CON: basal diet; CA: basal diet + 40 mg/kg of carnosic acid.

According to the above results, the cecal content of birds in the CON and CA40 groups were used for microbiota analysis. As shown in Figure 3A, the birds with CA supplementation featured a higher α-diversity characterized by increased Ace, Chao, and Sobs indices (*p* < 0.05). Venn diagrams (Figure 3B) indicated that the number of OTUs in the CA supplementation group was higher than that in the control group at the phylum, class, order, family, and genus levels. In addition to the nine phyla shared with the control group, three unique phyla (Synergistota, Deferribacterota, and Patescibacteria) were detected in the CA supplementation group.

**Figure 4:**
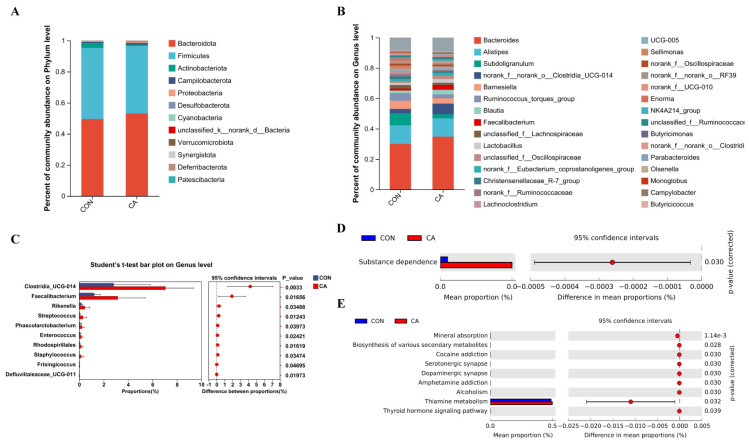
Effect of carnosic acid on differential microorganisms of the cecum. The microbial composition in cecal contents at the phylum (**A**) and genus (**B**) levels. Differences in microbes in the cecal between the CON and CA groups at the genus (**C**) levels. PICRUSt2 combined with the KEGG database to predict the function of bacterial microbiota, showing the results of the KEGG pathway in Level 2 (**D**) and Level 3 (**E**). CON: basal diet; CA: basal diet + 40 mg/kg carnosic acid. * *p* < 0.05. ** *p* < 0.01.

As shown in Figure 4A, microbiota profiling revealed that the phyla Bacteroidota and Firmicutes dominated the bird cecal contents. Among them, mainly genus *Bacteroides* and *Alistipes* accounted for about 40% (Figure 4B). *Clostridia_UCG-014*, *Faecalibacterium*, *Rikenella*, *Streptococcus*, *Phascolarctobacterium*, *Enterococcus*, and *Bacteria* were significantly (*p* < 0.05) enriched in the CA group (Figure 4C). As shown in Figure 4D,E, PICRUSt2 showed that CA supplementation enriched (*p* < 0.05) the substance dependence signaling pathway at the Level 2 KEGG pathway, and mineral absorption, biosynthesis of various secondary metabolites-part 3, cocaine addiction, serotonergic synapse, dopaminergic synapse, amphetamine addiction, alcoholism, thiamine metabolism, and thyroid hormone signaling pathway at the Level 3 KEGG pathway.

## 4. Discussion

There are few studies on the effect of CA on broiler chicken growth performance; however, there are relevant studies demonstrating the growth-promoting potential of phenolic plant extracts in poultry production. For example, the supplementation with polyphenol-rich grape seed to the ration increased the final BW and ADG of Cobb-500 broilers and improved the feed conversion ratio [23]. In addition, dietary supplementation with 400 mg/kg resveratrol increased the ADG of white broilers, reduced the feed-to-weight ratio, and improved the growth performance; while high doses of resveratrol (1000 mg/kg) inhibited the growth and reduced the survival rate of broilers [24]. Furthermore, our previous research found that supplementation with CA increased the ADG of LPS-challenged broilers [14]. Similar results were shown in the current research, in which supplementation with 10, 20, or 40 mg/kg of CA increased the final BW and ADG of broilers at the later phase, and the survival rate was significantly reduced when the CA supplementation level reached 80 mg/kg. Moreover, 80 mg/kg of CA significantly reduced the relative weight of the liver, indicating that a high concentration of CA might damage liver cells and their functions, lead to abnormal liver metabolism, and thereby affect the production performance of broiler chickens. The above together suggested that, while appropriate doses of plant polyphenols might enhance growth performance, excessive amounts could have adverse effects.

Although CA supplementation showed no significant effects on pectoral muscle yield and meat quality, the CA40 treatment exhibited a tendency to reduce IMF content, which mainly refers to the lipids located in skeletal muscle tissue, and is an important indicator for evaluating meat quality [25]. This suggested that CA might improve meat quality to a certain extent, although the effect was limited.

Intestinal villi increased the contact surface area for promoting nutrient absorption, while a shallower crypt depth indicated the higher maturation rate of intestinal epithelial cells and the greater absorptive capacity [26,27]. In the current research, supplementation with CA increased the V/C of the jejunum. For LPS-challenged broilers in our previous research, supplementation with CA increased the villus height to crypt depth ratio and elevated the relative expression of *Claudin-1/-2* and *ZO-1/-2/-3* in intestine, strengthening the tight junction in intestinal epithelial cells of the duodenum [14]. Polyphenols have demonstrated positive effects on intestinal morphology and mucosal development. For instance, magnolol increased the duodenal and the ileal villus height [28], as well as the duodenal wall thickness [29]. Therefore, CA might potentially enhance nutrient absorption and growth performance by increasing the intestinal V/C and improving intestinal health.

The presence of numerous stressors (feed, environment, etc.) in the intensive broiler farming model could cause peroxidation in livestock [30,31], causing damage to the intestines of the broiler chickens, which consequently reduced the feed conversion efficiency of the broiler chickens and affected their production performance [32,33]. Adding moderate amounts of antioxidant supplements to feed could serve as a safe and effective nutritional regulation strategy to enhance the capacity of the antioxidant system [34]. The content of lipid peroxides (such as MDA) reflected the level of oxidative stress, and their excessive accumulation could cause further damage to cells and tissues [35,36]. By activating the enzymatic antioxidant system and by enhancing the activities of enzymes such as GSH-Px, T-AOC, T-SOD and CAT, the clearance of reactive oxygen species (ROS) could be accelerated, the chain reaction of lipid peroxidation could be blocked, and the content of MDA could be reduced [37,38,39]. The antioxidant properties of CA have been confirmed in multiple in vitro studies [40,41]. Poultry research has further supported these findings, with 40 mg/kg of CA supplementation reducing serum MDA levels in LPS-stressed AA broilers [11], and rosemary leaf powder (main ingredient was CA) increasing activities of jejunal SOD and CAT in laying hens [16]. Previous findings were consistent with the results of the current study, in which CA reduced the MDA levels in the plasma and liver of broiler chickens, while increasing the activities of hepatic T-SOD, as well as ileal GSH-Px and CAT, demonstrating a clear dose-dependent response. Supplementation with 20 and 40 mg/kg of CA showed most antioxidant effects, whereas a higher dose of 80 mg/kg of CA showed reduced efficacy, indicating an upper limit for beneficial antioxidant effects.

As a pivotal transcription factor governing redox homeostasis in cellular oxidative stress responses, Nrf2 upregulated the expression and biosynthesis of antioxidant enzymes, thereby efficiently scavenging free radicals to reduce oxidative damage and improve intestinal function [42,43]. *HO-1*, *GSR*, and *GSH-Px* acted as downstream targets of *Nrf2* to directly enhance antioxidant capacity through distinct mechanisms: HO-1 mediated the heme metabolism to generate antioxidant byproducts (e.g., bilirubin) [44], GSR maintained reduced GSH levels by regenerating GSH from GSSG [45], and GSH-Px catalyzed free radical scavenging using GSH as a co-substrate [46]. In vitro studies have demonstrated that CA (1 μM, 12 h) promoted GSH synthesis by decreasing SOD and increasing γ-glutamylcysteine ligase (γ-GCL) activity in SH-SY5 Y cells [47,48]. In vivo evidence has showed that supplementation with 30 or 60 mg/kg (BW) of CA improved both non-enzymatic (GSH) and enzymatic (GSH-Px, SOD, and CAT) antioxidant system and decreased MDA levels in mouse eye and brain tissues [49]. Treatment with CA reduced oxidative damage in mice with traumatic brain injury by activating the Nrf2–ARE signaling pathway [50]. In the current research, supplementation with 40 mg/kg of CA increased the expression of antioxidant-related genes (e.g., *Nrf2*, *HO-1*, *GSR*, *GSH-Px*), while also decreasing the *Keap1* expression of the broilers, indicating the activated Nrf2–Keap1 signaling axis enhances the cellular antioxidant defenses of broilers. As an exogenous antioxidant, CA protected the intestinal health and improved the growth performance in broilers by downregulating the expression of *Keap1* and activating *Nrf2*, primarily by activating antioxidant enzyme genes to enhance antioxidant enzyme activity, and thus protecting the intestinal health of broilers and improving their growth performance.

The intestinal microbiota was an important factor affecting poultry health and production, and its homeostasis played a significant role in intestinal function. In this study, supplementation with CA increased the α-diversity of the cecal microbiota, as evidenced by the increased Ace, Chao, and Sobs indices, indicating a more complex and diverse microbial ecosystem. The elevation in microbial diversity was associated with improved intestinal health and resistance against pathogen invasion [51]. This observation was further supported by a Venn diagram analysis, revealing that the CA group contained more OTUs at different taxonomic levels, which indicated that CA supplementation promoted a richer and more diverse microbiota composition. Notably, three bacterial phyla were uniquely present in the CA group, while absent in the CON group, demonstrating the distinct microbial profile induced by CA. These results indicated that CA might facilitate either the introduction or proliferation of beneficial microbes, thereby contributing to the homeostasis and health of the gut environment [52].

Microbiota analyses revealed that Bacteroidota and Firmicutes were the dominant phyla in the cecum contents of broilers, consistent with previous findings [53]. Notably, the abundances of beneficial genera (e.g., *Clostridia_UCG-014*, *Faecalibacterium*, *Rikenella*, *Phascolarctobacterium*) were significantly increased in the CA group. These genera had been associated with the production of short-chain fatty acids (SCFAs), particularly butyrate and propionate [54,55], suggesting that CA might enhance intestinal barrier function, mitigate inflammation, and improve antioxidant capacity through the beneficial modulation of SCFA production [56,57]. *Streptococcus* and *Enterococcus* were often considered commensal bacteria, and their increase further suggested that the microbial community in birds receiving CA was in balance to compete with pathogenic bacteria and maintain intestinal homeostasis [58]. Functional analyses using PICRUSt2 showed that CA supplementation significantly enriched various KEGG pathways, including those related to substance dependence signaling and metabolic processes. Enrichment of the pathways for mineral absorption, biosynthesis of various secondary metabolites, and thiamine metabolism was shown to promote nutrient metabolism and absorption, thereby further maintaining broiler gut health and growth performance [59]. Enrichment of the pathways related to substance dependence—including cocaine addiction, serotonergic synapse, and dopaminergic synapse—might be associated with the gut–brain axis, whereby gut microbiota influence brain function and behavior [60,61]. Emerging evidence suggested that the microbiota-mediated regulation of the gut–brain axis contributed to the alleviation of oxidative stress [62,63]. However, the specific role of antioxidant CA on the microbiota–gut–brain axis required further investigation.

## 5. Conclusions

Supplementation with CA enhanced the antioxidant capacity of broilers, increased the diversity and abundance of gut microbiota, and thus protected the intestinal health and improved the growth performance in broilers. The enhancing effect on antioxidant capacity is speculated to be related to the regulation of the Keap1/Nrf2 signaling pathway. However, this mechanism of action still requires further clarification of the causal relationship in subsequent experiments.

Under the conditions of this experiment, quadratic regressions for different variables showed that the optimal range for supplemental CA in the chicken’s diet was 19.11~76.85 mg/kg. Combined with experimental observation and regression analysis, the optimal level of supplementation was 40 mg/kg.

## Figures and Tables

**Table 1:** Composition and nutrient content of diets (air dry-basis).

Items	1 to 21 Days of Age	22 to 53 Days of Age
Ingredient, %		
Corn	56.50	63.40
Soybean meal	35.90	30.40
Soybean oil	2.60	1.50
*L*-Lys HCl (78.8%)	0.20	0.20
*DL*-Met (99.0%)	0.30	0.30
Talcum powder	1.00	1.00
Dicalcium phosphate	2.20	1.90
NaCl	0.30	0.30
Premix ^1^	1.00	1.00
Total	100.00	100.00
Nutrient levels ^2^		
Nitrogen-corrected metabolizable energy, MJ/kg	11.92	12.34
Crude protein, %	20.94	17.81
Calcium, %	1.01	0.92
Total phosphorus, %	0.72	0.67
Non-phytate phosphorus, %	0.47	0.41
Lysine, %	1.29	1.15
Methionine, %	0.52	0.48
Methionine + Cysteine, %	0.93	0.85

^1^ The premix provided the following per kilogram of diets during 1 to 21 days of age: VA 15,000 IU, VD_3_ 3300 IU, VE 20 IU, VK_3_ 6.00 mg, VB_1_ 1.80 mg, VB_2_ 9.00 mg, VB_6_ 3.50 mg, VB_12_ 0.01 mg, choline chloride 500.00 mg, niacin 60.00 mg, pantothenic acid 16.00 mg, folic acid 0.55 mg, biotin 0.15 mg, Fe 80.00 mg, Cu 8.00 mg, Mn 80.00 mg, Zn 60.00 mg, I 0.35 mg, and Se 0.30 mg. The premix provided the following per kilogram of diets during 22 to 53 days of age: VA 15,000 IU, VD_3_ 3300 IU, VE 20 IU, VK_3_ 6.00 mg, VB_1_ 3.00 mg, VB_2_ 9.00 mg, VB_6_ 6.00 mg, VB_12_ 0.03 mg, choline chloride 1000.00 mg, niacin 60.00 mg, pantothenic acid 18.00 mg, folic acid 0.75 mg, biotin 0.10 mg, Fe 80.00 mg, Cu 12.00 mg, Mn 100.00 mg, Zn 75.00 mg, I 0.35 mg, and Se 0.15 mg. ^2^ Values were calculated according to Nutrient Requirements of Yellow Chickens [20], and values of crude protein, calcium and total phosphorus were determined by analysis.

**Table 2:** Primer sequences for Real-time PCR.

Gene	Primer Sequence (5′ to 3′)	GenBank ID
*β-actin*	F: GAGAAATTGTGCGTGACATCA R: CCTGAACCTCTCATTGCCA	NM_001101.5
*GST*	F: GATGAACGTCGTCCAACCAG R: TCATGTCCGTGGTCCTTCAA	NM_001001777.2
*SOD1*	F: GGTGCTCACTTTAATCCTG R: CTACTTCTGCCACTCCTCC	NM_205064.2
*SOD2*	F: TGGTGTTCAAGGATCAGGCT R: CCCAGCAATGGAATGAGACC	NM_204211.2
*GSR*	F: ATACCCGGCGTCAGGTTTAA R: CCTGTCGCAATGAGGATGTG	XM_040671422.2
*Keap1*	F: CAACTTCGCCGAGCAGA R: CGTGGAACACCTCCGACT	XM_040671565.2
*HO-1*	F: CTTCGCACAAGGAGTGTTAAC R: CATCCTGCTTGTCCTCTCAC	NM_205344.2
*Nrf2*	F: ATCACCTCTTCTGCACCGAA R: GCTTTCTCCCGCTCTTTCTG	XM_046921122.1
*GSH-Px*	F: AAGTGCCAGGTGAACGGGAAGG R: AGGGCTGTAGCGGCGGAAAG	NM_001277854.3

F: forward primer; R: reverse primer; *GST*: glutathione s-transferase; *SOD1*: superoxide dismutase 1; *SOD2*: superoxide dismutase 2; *GSR*: glutathione reductase; *Keap1*: kelch-like ECH-associated protein-1; *Nrf2*: nuclear factor erythroid 2-related factor 2; *HO-1*: heme oxygenase-1; *GSH-Px*: glutathione peroxidase.

**Table 3:** Effects of carnosic acid on growth performance of broilers.

Items	CON	CA5	CA10	CA20	CA40	CA80	SEM	*p*-Value	Linear	Quadratic
1 to 21 days of age										
Initial BW, g	40.90	40.83	40.83	40.87	40.92	40.88	0.03	0.266	0.340	0.552
Final BW, g	468.67	462.33	461.50	464.50	470.00	465.00	4.24	0.816	0.789	0.901
ADG, g	21.39	21.07	21.03	21.18	21.45	21.21	0.21	0.814	0.789	0.905
ADFI, g	36.17	35.54	35.93	35.67	35.53	35.26	0.34	0.661	0.141	0.329
F/G	1.68	1.70	1.69	1.69	1.66	1.66	0.01	0.122	0.017	0.057
Survival rate, %	100.00	100.00	98.81	100.00	100.00	97.62	0.71	0.186	0.043	0.064
22 to 53 days of age										
Final BW, g	2013.06 ^b^	2056.04 ^ab^	2077.70 ^a^	2085.06 ^a^	2074.50 ^a^	2040.36 ^ab^	15.21	0.019	0.980	0.012
ADG, g	48.18 ^b^	49.77 ^a^	50.37 ^a^	50.52 ^a^	49.98 ^a^	49.15 ^ab^	0.52	0.044	0.914	0.049
ADFI, g	119.19	120.05	121.92	122.23	121.16	118.69	1.47	0.618	0.501	0.256
F/G	2.46	2.42	2.42	2.41	2.48	2.39	0.02	0.122	0.427	0.292
Survival rate, %	100.00 ^a^	98.61 ^a^	98.61 ^a^	100.00 ^a^	98.61 ^a^	90.15 ^b^	1.68	<0.001	<0.001	<0.001
1 to 53 days of age										
ADG, g	35.86 ^b^	36.64 ^ab^	37.03 ^a^	37.17 ^a^	36.97 ^a^	36.35 ^ab^	0.37	0.019	0.979	0.011
ADFI, g	90.09	90.90	92.44	91.99	91.58	90.12	1.38	0.640	0.088	0.007
F/G	2.51	2.48	2.50	2.48	2.53	2.45	0.03	0.171	0.216	0.107
Survival rate, %	100.00 ^a^	98.61 ^a^	95.83 ^a^	100.00 ^a^	98.61 ^a^	88.89 ^b^	1.88	<0.001	<0.001	<0.001

^ab^ Means with common superscripts within a row do not differ significantly (*p* ≥ 0.05). CON: basal diet; CA5/10/20/40/80: basal diet supplemented with 5, 10, 20, 40, and 80 mg/kg of carnosic acid; BW: body weight; ADG: average daily gain; ADFI: average daily feed intake; F/G: feed to gain ratio.

**Table 4:** Effects of carnosic acid on relative weights of immune organs of broilers.

Items	CON	CA5	CA10	CA20	CA40	CA80	SEM	*p*-Value	Linear	Quadratic
21 days of age										
Liver/BW	3.27	3.25	2.94	3.15	3.28	3.35	0.12	0.728	0.112	0.245
Spleen/BW	0.17	0.17	0.14	0.16	0.16	0.17	0.01	0.670	0.703	0.390
Thymus/BW	0.48	0.38	0.42	0.41	0.44	0.43	0.03	0.330	0.936	0.537
Bursa of Fabricius/BW	0.27	0.25	0.23	0.24	0.25	0.25	0.02	0.307	0.287	0.568
53 days of age										
Liver/BW	2.29 ^ab^	2.24 ^bc^	2.37 ^a^	2.26 ^b^	2.28 ^ab^	2.14 ^c^	0.02	0.010	0.002	0.003
Spleen/BW	0.22	0.23	0.21	0.22	0.22	0.20	0.01	0.380	0.059	0.160

^a–c^ Means with common superscripts within a row do not differ significantly (*p* ≥ 0.05). CON: basal diet; CA5/10/20/40/80: basal diet supplemented with 5, 10, 20, 40, and 80 mg/kg of carnosic acid; BW: body weight.

**Table 5:** Effects of carnosic acid on meat quality and IMF content of 53-day-old broilers.

Items	CON	CA5	CA10	CA20	CA40	CA80	SEM	*p*-Value	Linear	Quadratic
Pectoral muscle rate, %	12.67	12.42	12.23	12.58	12.63	12.85	0.14	0.195	0.059	0.103
Shear force_24h_, N	22.55	24.32	24.73	23.70	24.22	26.42	0.88	0.894	0.176	0.207
Drip loss_24h_, %	1.83	1.63	1.69	1.70	1.55	1.57	0.04	0.396	0.207	0.176
a*_45min_	13.94	13.54	13.87	14.00	13.30	13.48	0.10	0.268	0.298	0.556
b*_45min_	9.75	9.95	9.97	9.82	9.69	9.50	0.11	0.851	0.245	0.508
L*_45min_	49.95	50.29	50.31	49.81	50.57	49.76	0.18	0.771	0.775	0.822
pH_45min_	6.13	6.08	6.05	5.95	6.19	6.16	0.03	0.123	0.264	0.527
a*_24h_	13.92	13.58	13.19	13.18	13.17	13.37	0.23	0.928	0.550	0.621
b*_24h_	10.10	10.11	10.26	10.46	10.02	10.21	0.13	0.952	0.928	0.995
L*_24h_	55.03	53.31	54.46	54.72	54.28	54.22	0.29	0.658	0.784	0.683
pH_24h_	5.65	5.68	5.65	5.66	5.67	5.64	0.01	0.855	0.176	0.207
Intramuscular fat, %	8.51 ^a^	8.19 ^a^	8.66 ^a^	8.13 ^ab^	3.78 ^b^	8.62 ^a^	0.82	0.001	0.340	0.001

^ab^ Means with common superscripts within a row do not differ significantly (*p* ≥ 0.05). CON: basal diet; CA5/10/20/40/80: basal diet supplemented with 5, 10, 20, 40, and 80 mg/kg of carnosic acid; a* = redness; b* = yellowness; L* = lightness.

**Table 6:** Effects of carnosic acid on antioxidant capacity of 21-day-old broilers.

Item	CON	CA5	CA10	CA20	CA40	CA80	SEM	*p*-Value	Linear	Quadratic
Plasma										
MDA, nmol/mL	2.49	2.34	2.39	2.50	2.70	2.59	0.11	0.253	0.499	0.752
GSH-Px, U/mL	1491.86 ^bc^	1548.84 ^b^	1439.53 ^c^	1405.81 ^c^	1469.77 ^bc^	1768.6 ^a^	19.02	<0.001	<0.001	<0.001
T-SOD, U/mL	164.19 ^a^	163.66 ^a^	164.73 ^a^	164.53 ^a^	164.06 ^a^	162.05 ^b^	0.19	<0.001	0.014	<0.001
T-AOC, U/mL	1.34 ^c^	1.37 ^c^	2.14 ^ab^	1.22 ^c^	1.62 ^bc^	2.41 ^a^	0.27	0.004	0.004	0.011
Liver										
MDA, nmol/mg prot	0.29 ^a^	0.22 ^ab^	0.24 ^a^	0.21 ^ab^	0.21 ^ab^	0.13 ^b^	0.01	0.038	0.002	0.130
GSH-Px, U/mg prot	75.47	82.89	84.19	77.66	76.72	74.49	1.49	0.313	0.172	0.390
T-SOD, U/mg prot	23.24	24.89	24.88	24.38	24.08	23.56	0.42	0.480	0.424	0.717
T-AOC, U/mg prot	0.13 ^ab^	0.13 ^ab^	0.14 ^a^	0.13 ^ab^	0.12 ^bc^	0.11 ^c^	0.02	0.002	0.001	0.004
CAT, U/mg prot	74.72 ^b^	82.92 ^a^	82.22 ^a^	76.29 ^ab^	76.43 ^ab^	75.11 ^b^	1.01	0.048	0.070	0.155
Duodenum										
MDA, nmol/mg prot	0.89	0.70	0.71	0.73	0.96	0.99	0.09	0.069	0.023	0.075
GSH-Px, U/mg prot	12.90 ^c^	19.74 ^bc^	21.54 ^ab^	31.35 ^a^	23.61 ^ab^	30.51 ^a^	1.52	<0.001	0.002	0.002
T-SOD, U/mg prot	29.20 ^c^	38.94 ^a^	37.42 ^ab^	33.52 ^bc^	39.07 ^a^	40.34 ^a^	0.78	<0.001	0.001	0.003
T-AOC, U/mg prot	2.61 ^c^	3.83 ^a^	3.29 ^ab^	2.84 ^bc^	3.38 ^ab^	3.36 ^ab^	0.08	<0.001	0.414	0.678
CAT, U/mg prot	1.15	1.18	1.32	1.44	0.76	1.11	0.19	0.188	0.231	0.430
Jejunum										
T-SOD, U/mg prot	22.99 ^ab^	21.93 ^ab^	21.97 ^ab^	22.92 ^ab^	21.54 ^b^	23.59 ^a^	0.20	0.047	0.185	0.084
GSH-Px, U/mg prot	119.54	111.93	137.00	119.48	108.26	131.58	3.37	0.093	0.731	0.394
T-AOC, U/mg prot	0.24	0.22	0.23	0.24	0.21	0.25	0.01	0.164	0.281	0.318
Ileum										
MDA, nmol/mg prot	0.11	0.13	0.14	0.17	0.12	0.12	0.02	0.289	0.602	0.424
T-SOD, U/mg prot	8.52	7.86	7.83	8.41	8.24	7.77	0.28	0.246	0.307	0.466
GSH-Px, U/mg prot	14.22 ^b^	28.51 ^a^	23.49 ^a^	24.15 ^a^	25.24 ^a^	9.80 ^b^	1.18	<0.001	0.002	<0.001
T-AOC, U/mg prot	0.15 ^abc^	0.16 ^a^	0.15 ^ab^	0.15 ^abc^	0.13 ^bc^	0.13 ^c^	1.17	0.009	<0.001	0.003
CAT	2.62	2.74	2.79	3.30	2.36	2.77	1.00	0.176	0.878	0.727

^a–c^ Means with common superscripts within a row do not differ significantly (*p* ≥ 0.05). CON: basal diet; CA5/10/20/40/80: basal diet supplemented with 5, 10, 20, 40, and 80 mg/kg of carnosic acid; MDA: malondialdehyde; GSH-Px: glutathione peroxidase; T-SOD: total superoxide dismutase; T-AOC: total antioxidant capacity; CAT: catalase.

**Table 7:** Effects of carnosic acid on antioxidant capacity of 53-day-old broilers.

Item	CON	CA5	CA10	CA20	CA40	CA80	SEM	*p*-Value	Linear	Quadratic
Plasma										
MDA, nmol/mL	9.40 ^a^	9.31 ^a^	8.17 ^a^	4.10 ^b^	3.71 ^b^	4.26 ^b^	1.76	0.027	0.007	0.003
GSH-Px, U/mL	1376.60	1379.49	1355.69	1360.74	1386.70	1329.01	40.42	0.940	0.319	0.486
T-SOD, U/mL	151.32	152.92	149.89	147.19	129.51	130.52	7.70	0.085	0.002	0.006
T-AOC, U/mL	6.18	6.23	6.54	6.51	5.84	6.34	0.36	0.798	0.766	0.900
Liver										
MDA, nmol/mg prot	1.36 ^a^	0.82 ^b^	0.80 ^b^	0.75 ^bc^	0.55 ^c^	0.70 ^bc^	0.11	<0.001	0.001	<0.001
GSH-Px, U/mg prot	1436.44	1551.88	1626.89	1389.36	1712.59	1392.63	124.57	0.349	0.675	0.391
T-SOD, U/mg prot	577.78 ^b^	576.59 ^b^	584.04 ^b^	579.68 ^ab^	607.88 ^a^	609.67 ^a^	8.06	0.001	<0.001	<0.001
T-AOC, U/mg prot	2.46	2.46	2.68	2.20	2.56	2.88	0.17	0.092	0.064	0.110
CAT, U/mg prot	4.72 ^ab^	4.80 ^a^	4.80 ^a^	4.72 ^ab^	4.56 ^b^	4.58 ^b^	0.06	0.007	0.001	0.052
Duodenum										
MDA, nmol/mg prot	0.66	0.49	0.72	0.52	0.56	0.64	0.08	0.326	0.995	0.760
GSH-Px, U/mg prot	2302.82	3340.94	2676.97	2824.52	4463.37	4510.80	758.35	0.182	0.019	0.051
T-SOD, U/mg prot	825.25	825.74	806.00	770.15	755.95	762.21	27.94	0.273	0.042	0.051
T-AOC, U/mg prot	21.43	18.43	18.50	19.06	19.67	19.91	0.39	0.260	0.066	0.086
CAT, U/mg prot	1.15	1.18	1.32	1.44	0.76	1.11	0.19	0.183	0.271	0.469
Jejunum										
MDA, nmol/mg prot	1.36 ^a^	0.57 ^b^	0.61 ^ab^	0.70 ^ab^	0.64 ^a^	0.31 ^b^	0.29	0.048	0.039	0.066
GSH-Px, U/mg prot	35.69	36.62	43.24	43.05	33.87	36.71	4.56	0.638	0.653	0.896
T-SOD, U/mg prot	20.46	20.61	20.32	20.55	19.92	20.84	0.52	0.894	0.893	0.680
T-AOC, U/mg prot	0.25	0.25	0.28	0.29	0.27	0.27	0.02	0.488	0.180	0.279
CAT, U/mg prot	3.76	3.72	3.56	4.05	3.05	3.80	0.47	0.785	0727	0.800
Ileum										
MDA, nmol/mg prot	0.96	1.09	1.07	0.98	0.91	1.04	0.07	0.564	0.619	0.651
GSH-Px, U/mg prot	21.50 ^c^	45.26 ^a^	47.88 ^a^	41.02 ^ab^	27.09 ^c^	32.35 ^bc^	4.62	<0.001	0.298	0.471
T-SOD, U/mg prot	15.45	15.56	15.32	15.31	16.12	15.14	0.30	0.464	0.896	0.243
T-AOC, U/mg prot	0.24	0.25	0.25	0.25	0.27	0.24	0.01	0.581	0.825	0.070
CAT, U/mg prot	2.39 ^c^	2.57 ^bc^	3.11 ^bc^	3.58 ^ab^	2.80 ^bc^	3.84 ^a^	0.30	0.009	0.025	0.062

^a–c^ Means with common superscripts within a row do not differ significantly (*p* ≥ 0.05). CON: basal diet; CA5/10/20/40/80: basal diet supplemented with 5, 10, 20, 40, and 80 mg/kg of CA; MDA: malondialdehyde; GSH-Px: glutathione peroxidase; T-SOD: total superoxide dismutase; T-AOC: total antioxidant capacity; CAT: catalase.

**Table 8:** Regression analysis of the optimal supplemental level of carnosic acid for broilers based on quadratic regression.

Item	*p*-Value	*R* ^2^	Regression Equation	CA Supplemental Level (mg/kg) ^1^
Final BW (53 d), g	0.012	0.243	y = −0.0356117 CA^2^ + 2.88631 CA + 2034.329	40.53
ADG (22 to 53 d), g	0.049	0.171	y = −0.0010260 CA^2^ + 0.08236 CA + 49.211	40.14
ADG (1 to 53 d), g	0.011	0.244	y = −0.006492 CA^2^ + 0.05261 CA + 36.244	40.52
21 d				
Hepatic T-AOC, U/mg prot	0.004	0.160	y = −0.0000020 CA^2^ + 0.00009 CA + 0.130	24.17
Duodenal T-SOD, U/mg prot	0.003	0.188	y = −0.0012707 CA^2^ + 0.18963 CA + 33.018	74.61
Duodenal GSH-Px, U/mg prot	0.002	0.208	y = −0.0042395 CA^2^ + 0.50918 CA + 15.977	60.05
Ileal GSH-Px, U/mg prot	<0.001	0.407	y = −0.0067097 CA^2^ + 0.41349 CA + 19.535	30.81
53 d				
Relative weight of liver, %	0.003	0.165	y = −0.0000429 CA^2^ + 0.00164 CA + 2.281	19.11
Intramuscular fat, %	0.001	0.432	y = −0.0022318 CA^2^ + 0.19212 CA + 9.439	43.04
Jejunal V/C	0.036	0.143	y = −0.0003450 CA^2^ + 0.03950 CA + 5.169	57.28
Plasma MDA, nmol/mL	0.003	0.234	y = 0.0023638 CA^2^ − 0.26109 CA +10.047	55.23
Hepatic MDA, nmol/mg prot	<0.001	0.483	y = 0.0002654 CA^2^ − 0.02685 CA + 1.161	50.58
Hepatic T-SOD, U/mg prot	<0.001	0.329	y = −0.0075841 CA^2^ + 1.08413 CA + 566.677	71.47
Hepatic *Keap1*, relative expression	0.009	0.293	y = 0.0003332 CA^2^ − 0.0300 CA + 1.194	45.02
Hepatic *Nrf2*, relative expression	0.018	0.215	y = −0.0001084 CA^2^ + 0.00962 CA + 1.044	44.35
Hepatic *HO-1*, relative expression	0.012	0.235	y = −0.00004167 CA^2^ + 0.00640 CA + 1.034	76.85
Hepatic *GSH-Px*, relative expression	0.011	0.345	y = −0.0003055 CA^2^ + 0.037626 CA + 1.133	61.58

^1^ CA was supplemental level of carnosic acid (mg/kg), and y was the dependent variable. BW: body weight; ADG: average daily gain; T-AOC: total antioxidant capacity; T-SOD: total superoxide dismutase; GSH-Px: glutathione peroxidase; MDA: malondialdehyde; V/C: the ratio of villus height to crypt depth; *Keap1*: kelch-like ECH-associated protein-1; *Nrf2*: nuclear factor erythroid 2-related factor 2, *HO-1*: heme oxygenase-1; *GSR*: glutathione reductase.

## Data Availability

The original contributions presented in this study are included in the article. Further inquiries can be directed to the corresponding authors.
